# The prevalence of accessory renal arteries in sudanese population in Khartoum State: a cross-sectional CT study from 2017 to 2020

**DOI:** 10.1186/s12882-024-03573-3

**Published:** 2024-04-15

**Authors:** Safaa. Mohammed, Eltayeb. AbdAlla, Amal. Elhag, Abdelmoniem. El-Mardi

**Affiliations:** 1 Nursing Department, Faculty of Medical Sciences and Nursing, Alrayan Colleges, Almadina, Saudi Arabia; 2 Faculty of Medicine, University of Khartoum, Khartoum, Sudan; 3 Assistant Professor of Human Anatomy, Faculty of Medicine and Surgery, Umm Al-Qura University, Makkah, Saudi Arabia; 4 Biomedical Sciences Department, Dubai Medical College for Girls, Dubai, UAE

**Keywords:** Accessory renal arteries, Renal arteries variation, Early dividing renal arteries

## Abstract

**Background:**

Renal artery variations are clinically significant due to their implications for surgical procedures and renal function. However, data on these variations in Sudanese populations are limited. This study aimed to determine the prevalence and characteristics of renal artery variations in a Sudanese population.

**Methods:**

A cross-sectional retrospective study was conducted in Khartoum state from October 2017 to October 2020. A total of 400 Sudanese participants who underwent abdominal CT scans were included. Data on demographic characteristics, kidney measurements, and renal vasculature were collected and analyzed using descriptive statistics and inferential tests.

**Results:**

The mean age of participants was 46.7 ± 18 years, with a nearly equal gender distribution. Overall, renal artery variations were present in 11% of participants, with accessory renal arteries observed in 6% of the study population. Among those with accessory vessels, 50% were on the right side, 29.2% on the left, and 20.8% bilateral, distributed across hilar 29.2%, lower polar 29.2%, and upper polar 41.7% regions. No significant associations were found between accessory renal arteries and age or gender (*p*-value > 0.05). However, participants with accessory renal arteries exhibited significantly narrower width 5.0 ± 1.4 than those with no with accessory renal arteries 5.8 ± 1.1 (*p*-value 0.002) Early dividing renal arteries were found in 5% of participants, with nearly half being bilateral. No significant associations were found between the presence of early dividing renal arteries and demographic or renal measurements (*p*-value > 0.05).

**Conclusion:**

This study provides valuable insights into the prevalence and characteristics of renal artery variations in a Sudanese population. The findings contribute to our understanding of renal anatomy in this demographic and can inform clinical practice and surgical planning, particularly in renal transplantation and other renal procedures.

## Introduction

The renal arteries play a crucial role in supplying blood to the kidneys, accounting for approximately 20% of cardiac output. In most individuals, a single renal artery arises from the abdominal aorta, but approximately 30% exhibit accessory renal arteries (aRA) [1]. they originate from the abdominal aorta, common iliac, and superior mesenteric, vary in numbers as doubled, triplet, and four renal arteries have been reported [2–8]. These variants can have clinical implications, such as renal artery stenosis or complications during renal transplantation.

The prevalence of accessory renal arteries varies widely across different studies ranging from as low as 4% to as high as 61% [9–14]. Men exhibit a higher prevalence than women [15]. Moreover, there is a wide variation between ethnic groups [16–20].

The identification of additional renal arteries varies based on diagnostic methods, including cadaveric dissection or radiological imaging. Cadaver dissection provides precise results [9]. However, angiographic studies may miss accessory renal arteries due to their small diameter [21]. Nevertheless, three-dimensional CT angiography is highly effective in visualizing renal arteries and accessory arteries with 97.6% accuracy [22].

Accessory renal arteries have various clinical implications, including supplying blood to parts of the kidney [23, 24], affecting kidney transplantation outcomes, and influencing the risk of transplantation rejection [25]. They can also impact the size of adjacent vessels [16], and are associated with conditions like arterial thrombosis, elevated blood pressure, and renal artery stenosis [26, 27].

Accurate detection of accessory renal arteries is vital for surgical planning to avoid complications like bleeding or graft loss and for diagnosing certain kidney diseases, emphasizing the importance of understanding renal artery anatomy and variations for clinicians involved in diagnosis and surgical procedures [28–30].

Further investigation is needed to fully understand renal artery anomalies, especially in populations like Sudanese, where the prevalence of accessory renal arteries is understudied. This study aims to address this gap by assessing the prevalence of accessory renal arteries in a healthy Sudanese population using CT scans.

## Materials and methods

The study employed a cross-sectional retrospective design, spanning from October 2017 to October 2020, within the Khartoum state which contains Sudanese for all over Sudan and represent all the ethnic groups in Sudan. The study included individuals underwent abdominal CT scan in Dar Al Elaj Hospital, Ibn Sina Hospital, Ibn Al Hytham, and Ahmed Gasim Diagnostic Centers. To determine the sample size, a formula incorporating a desired margin of error, confidence level, and estimated prevalence of accessory renal arteries was utilized, resulting in approximately 385 patients [31].

Utilizing convenient non-probability sampling, individuals meeting inclusion criteria (All Sudanese adults who were indicated to undergo abdominal CT scan\) were recruited. Pregnant ladies, patients with diabetes, acute kidney injury, solitary kidney,, and those allergic to iodinatedcontrast media were excluded.

The study used CT abdomen examinations with a multi-detector CT scanner (Siemens Somatom Sensation 64 CT scan device) for renal artery identification. Participants underwent fasting for 6–8 h and received oral and intravenous contrast. The abdomen was scanned in the supine position from vertebral level T12 to the symphysis pubis, and renal arteries were detected in the arterial phase using a second run with a bolus tracking technique.

After CT angiography, images were processed using techniques like multi-planar reconstructions (MPR), maximum intensity projection (MIP), and volume rendering techniques (VRT) on the Advantage Windows 3D workstation. Arterial phase reconstruction was conducted with images reconstructed at a 1 mm slice thickness and 50% overlap.

Parameters evaluated from the CT images included the length and diameter of the main renal artery, the presence and number of accessory arteries, and the presence of early branching.

Data analysis was performed using SPSS version 28 software, with categorical data presented as frequencies and proportions, and continuous data represented as mean and standard deviation. Significance testing was conducted using chi-square and ANOVA tests, with a *p*-value of < 0.05 considered statistically significant.

## Results

The study included 400 Sudanese participants with a mean age of 46.7 ± 18 years, males were 202 (50.5%) and females were 189 (49.5%) with 1.02: 1 male to female ratio (Table 1).

**Table 1:** Demographic characteristics of Sudanese population underwent abdominal CT in Khartoum State (*n* = 400)

	Count (%) Mean ± SD	Accessory renal artery			Early dividing renal artery		
		No	Yes	*p*-value	No	Yes	*p*-value
Age	46.7 ± 18	46.6 ± 19	47.8 ± 15	0.758	46.7 ± 19	47.1 ± 14	0.926
Gender							
Male	202 (50.5%)	187 (92.6%)	15 (7.4%)	0.225	192 (95%)	10 (10%)	0.786
Female	189 (49.5%)	189 (95.5%)	9 (4.5%)		187 (94.4%)	11 (5.6%)	

Kidney and renal arteries measurements are shown in (Table 2) below. Overall renal arteries variation, were present in 44 patients (11%), Accessory renal artery was present in 24 participants constituting 6% of the study population; 50% (12/24) of these accessory vessels were in the right side, while 29.2% (7/24) were in the left side, and 20.8% (5/24) were bilateral. These vessels were either hilar 29.2% (7/24), lower polar 29.2% (7/24) or upper polar 41.7% (10/24).

Although the prevalence of accessory renal arteries was higher in males 7.4% (15/202) than females 4.5% (9/198) there was no statistically significant difference (*p*-value 0.225) (Table 1).

Although there was no significant difference between those with accessory renal arteries and those with no with accessory renal arteries regarding kidney measurements or renal arteries measurements (*p*-value > 0.05), participants with accessory renal arteries have significantly narrower width 5.0 ± 1.4 than those with no with accessory renal arteries 5.8 ± 1.1 (*p*-value 0.002) (Table 2).

Early dividing renal arteries were found in 5% (21/400) of study participants, which was bilateral in nearly half of them 47.6% (10/21), in the left side in 33.3% (7/21) and in the right side in 19% (4/21).

There was no significant relationship between the presence of early dividing renal arteries and gender, kidney measurements, nor renal arteries measurements (*p*-value > 0.05) (Tables 1 and 2).

**Table 2:** Relationship between kidney size and renal artery measures with the presence of accessory renal arteries among Sudanese population underwent abdominal CT in Khartoum State (*n* = 400)

Variable	Measure	Accessory renal artery			Early dividing renal artery			Total
		No	Yes	*p*-value	No	Yes	*p*-value	
Right kidney	Width	5.4 ± 0.8	5.3 ± 0.6	0.584	5.4 ± 0.8	5.2 ± 0.7	0.557	5.39 ± 0.8
	Length	10.0 ± 1	10.2 ± 1.2	0.438	10.0 ± 1	10.2 ± 0.9	0.537	10.1 ± 1
Left kidney	Width	5.6 ± 0.8	5.5 ± 0.7	0.633	5.6 ± 0.8	5.7 ± 0.8	0.517	5.6 ± 0.8
	Length	10.1 ± 1.2	10.4 ± 0.8	0.216	10.1 ± 1.2	10.3 ± 1.2	0.490	10.1 ± 1.2
Right renal artery	Width	5.8 ± 1.1	5.0 ± 1.4	0.002	5.8 ± 1.1	5.5 ± 1.4	0.441	5.78 ± 1.1
	Length	5.9 ± 1	5.8 ± 0.9	0.844	5.9 ± 1	6.0 ± 1.3	0.437	5.9 ± 1
Left renal artery	Width	5.9 ± 1.1	5.5 ± 1.2	0.067	5.9 ± 1.1	5.9 ± 1.2	0.897	5.9 ± 1.1
	Length	5.0 ± 0.8	5.0 ± 1.2	0.724	5.0 ± 0.8	5.0 ± 1.2	0.664	5.0 ± 0.9

## Discussion

Understanding the variations in renal arteries has become increasingly crucial given the rise in renal transplants, vascular reconstructions, and various surgical, urological, and radiological procedures performed in recent times. However, it’s noted that the prevalence and types of accessory renal arteries vary among populations. Thus, this study aimed to investigate the occurrence and anatomical variations of accessory renal arteries in the Sudanese population.

The study revealed that renal artery variations were present in 11% of the 400 Sudanese participants. This prevalence was lower than recent reports in Sudan, where it ranged from 20 to 30%, as found by Mustafa et al. (2016) and Salih et al. (2018) [32, 33]. Additionally, it was lower than previous reports worldwide which varied from 31 to 59.6% [34–37]. The study excluded a group of patients who cannot undergo Abdominal CT with contrast, this might have resulted in this lower rate of renal arteries variation, as many previous studies including those conducted in Sudan recruited cadaveric dissection for the detection of renal artery variations [32, 33].

These findings highlight the frequent occurrence of structural differences in renal arteries across diverse populations worldwide. The presence of such variations underscores the importance of conducting comprehensive preoperative imaging examinations, particularly in kidney donation and transplant procedures. Surgeons, armed with a better understanding of the prevalence and characteristics of these variations, can more effectively anticipate and address potential surgical challenges, ultimately leading to improved patient outcomes. Thus, it is imperative for medical practitioners to be cognizant of these variations to ensure the success of kidney donation and transplant procedures.

Accessory renal arteries were present in 6% of study population Previous local reports indicated that the prevalence of accessory renal arteries in Sudanese population ranged from 20 to 25% [32, 33]. Although the frequency reportedly varies among different populations, ranging from 4% of Malaysian patients to 61.5% of Brazilian patients [38], consensus with recent reports emphasizes that they are the most common renal artery variation [37, 39]. Early dividing renal arteries however, were found in 5% of the Sudanese subjects. Comparable to the reported prevalence in other populations which ranged between 6.5% in dd [40] and Sudanese [33] to 20.2% in Indians [37].

The discrepancies in these findings underscore the need for further research to better understand the prevalence and distribution of accessory renal arteries. Differences in findings may be attributed to factors such as sample size, population demographics, and methodology utilized in each study. It is suggested that cadaver dissection may provide a more accurate assessment of the number of renal arteries compared to aortography [33]. Additionally, accessory renal arteries are less frequently detected in angiographic studies due to their small thickness, which may explain the lower prevalence observed in this study compared to previous studies. However, it is emphasized that the arterial phase of three-dimensional CT angiography is highly sensitive in detecting renal arteries and other arterial abnormalities [22].

Whether accessory renal arteries are prevalent on one side is controversial, some studies report that accessory renal arteries are most frequently left-sided such as Costa et al. (2011) [41], while others find that the right side predominates such as Holden et al. (2005) [42]. In this study, right side accessory renal artery was the most prevalent. Similarly, their entry into the kidney, while Guan et al. (2013) indicated the inferior pole [43], Bordei et al. indicate that the hilum [44] as the commonest root of entry. Conversely, the present study showed that the upper pole was the most common root of entry among the Sudanese population. Moreover, it is uncertain whether sex affects the occurrence of accessory renal arteries or not, the present study found no statistically significant difference, supporting Çlnar et al. (2016) who reported no statically significant difference between males and females [40]. However, statistically significant higher incidence in males than females was reported by Tardo et al. (2017) [39].

The implications of accessory renal arteries are not fully understood or categorized, as they are typically discovered incidentally during pre-operative procedures or diagnostic evaluations. However, during surgical procedures, the presence of upper pole arteries poses a significant risk due to their proximity to the kidney. Surgeons may inadvertently cut through these arteries, mistaking them for surrounding tissue, leading to severe bleeding and potentially fatal consequences [9].

An important factor associated with accessory renal arteries is renal volumetry, with recent studies suggesting a direct relationship between the presence of accessory renal arteries and total renal volume, as well as an inverse relationship with the diameter of the main renal artery [9]. Similarly, the present study found that participants with accessory renal arteries had significantly narrower renal artery diameters compared to those without. This finding is consistent with previous research by Aytac et al. (2003), who noted that a smaller-than-usual diameter of the main renal artery in a kidney with normal dimensions may indicate the presence of an accessory renal artery [45].

Addressing that accessory renal arteries may influence the diameter of the main renal artery suggests a potential role for these additional vessels in renal vascular dynamics. Additionally, Rizzari et al. highlight that individuals with kidneys harboring two or more arteries exhibit a heightened prevalence of hypertension, possibly attributable to the narrowing of the renal arteries [46]. However, further investigation is warranted to delve into the clinical ramifications of these variations in renal artery measurements and their impact on overall renal function and cardiovascular health.

The present study poses a significant value of adding to the body of literature the prevalence of accessory renal arteries in the Sudanese population, additionally, the study included subjects from diverse centers further strengthening the generalizability of the findings. However, it worth mentioning some limitations, such as the reliance solely on CT scans for renal artery identification might have overlooked certain variations that could be better detected through other diagnostic methods such as cadaveric dissection. Furthermore, the study did not account for potential ethnic diversity within the Sudanese population, which could impact the prevalence and characteristics of renal artery variations.

The study reveals a significant presence of accessory renal arteries in the Sudanese population, highlighting the importance of understanding renal artery anatomy for surgical planning and diagnostics. The association between accessory renal arteries and narrower main renal artery diameters suggests potential implications for renal vascular dynamics and overall kidney function. Future research should recruit larger sample sizes, conduct comparative studies with different diagnostic methods, and explore longitudinal outcomes associated with accessory renal arteries. Ethnic diversity within Sudan is also crucial for understanding variations in renal artery anatomy.

## Data Availability

Data are available upon reasonable request from the corresponding author.

## Declarations

The study received ethical approval from the National University’s ethical committee. Then it was delivered to the Federal Ministry of Health (FMOH) and the administration of the participating centres. The need for informed consent was waived by the ethical committee of the Research and Training section at the Federal Ministry of Health (FMOH) due to the use of data obtained from medical records.

## Abbreviations

ANOVA: Analysis of Variance
aRA: Accessory Renal Arteries
CT: Computed Tomography
MIP: Maximum Intensity Projection
MPR: Multi-Planar Reconstructions
MRA: Magnetic Resonance Angiography
SD: Standard Deviation
SPSS: Statistical Package for the Social Sciences
VRT: Volume Rendering Techniques

## References

[1] Standring S, Ellis H, Healy J, Johnson D, Williams A, Collins P. Gray’s Anatomy. The Anatomical Basis of Clinical Practice [Internet]. Am J Neuroradiol; 2005 [cited 2024 Feb 15]. https://www.researchgate.net/publication/284761438_Gray’s_Anatomy_The_Anatomical_Basis_of_Clinical_Practice/citation/download

[2] Dhar P, Lal K (2005). Main and accessory renal arteries - a morphological study. Italian J Anat Embryol.

[3] Le Dorze M, Bouglé A, Deruddre S, Duranteau J. Renal doppler ultrasound: A new tool to assess renal perfusion in critical illness. Vol. 37, Shock. Shock; 2012. pp. 360–5. 10.1097/SHK.0b013e3182467156 22258233

[4] Shimada K, Ohashi I, Sakai Y, Kijima T, Yoshida S, Okuno T (2006). An unusual renal vascular anomaly: common origin of arteries to the lower poles demonstrated by a computed tomography angiography using 16-slice multidetector computed tomography. Acta Radiol.

[5] Asala SA, Masumbuko-Kahamba N, Bidmos MA (2001). An unusual origin of supernumerary renal arteries: Case report. East Afr Med J.

[6] Lacout A, Thariat J, Marcy PY. Main right renal artery originating from the superior mesenteric artery. Vol. 25, clinical anatomy. Clin Anat; 2012. pp. 977–8. 10.1002/ca.22002 22109639

[7] Gesase AP (2007). Rare origin of supernumerary renal vessels supplying the lower Pole of the left kidney. Annals Anat.

[8] Loukas M, Aparicio S, Beck A, Calderon R, Kennedy M (2005). Rare case of right accessory renal artery originating as a common trunk with the inferior mesenteric artery: a case report. Clin Anat.

[9] Gulas E, Wysiadecki G, Szymański J, Majos A, Stefańczyk L, Topol M, et al. Morphological and clinical aspects of the occurrence of accessory (multiple) renal arteries. Archives of Medical Science. Volume 14. Termedia Publishing House Ltd.; 2018. pp. 442–53. 10.5114/aoms.2015.55203 PMC5868651 29593819

[10] Rimoldi SF, Scheidegger N, Scherrer U, Farese S, Rexhaj E, Moschovitis A (2014). Anatomical eligibility of the renal vasculature for catheter-based renal denervation in hypertensive patients. JACC Cardiovasc Interv.

[11] Okada T, Pellerin O, Savard S, Curis E, Monge M, Frank M (2015). Eligibility for renal denervation: anatomical classification and results in essential resistant hypertension. Cardiovasc Intervent Radiol.

[12] Sato Y, Kawakami R, Jinnouchi H, Sakamoto A, Cornelissen A, Mori M (2021). Comprehensive Assessment of Human Accessory Renal Artery Periarterial Renal sympathetic nerve distribution. JACC Cardiovasc Interv.

[13] Glodny B, Cromme S, Wörtler K, Winde G (2001). A possible explanation for the frequent concomitance of arterial hypertension and multiple renal arteries. Med Hypotheses.

[14] Savard S, Frank M, Bobrie G, Plouin PF, Sapoval M, Azizi M (2012). Eligibility for renal denervation in patients with resistant hypertension: when enthusiasm meets reality in real-life patients. J Am Coll Cardiol.

[15] Satyapal KS, Haffejee AA, Singh B, Ramsaroop L, Robbs JV, Kalideen JM (2001). Additional renal arteries: incidence and morphometry. Surg Radiol Anat.

[16] Khamanarong K, Prachaney P, Utraravichien A, Tong-Un T, Sripaoraya K (2004). Anatomy of renal arterial supply. Clin Anat.

[17] TAO X feng ZHUJ, qi WUY, wei TANGG, yu SHIY, zhen ZHANGL et al. Dual-energy computed tomography angiography for evaluating the renal vascular variants. Chin Med J (Engl) [Internet]. 2013 Feb 20 [cited 2024 Feb 18];126(4):650–4. https://journals.lww.com/00029330-201302200-00009 23422183

[18] Johnson PB, Cawich SO, Shah SD, Aiken W, McGregor RG, Brown H (2013). Accessory renal arteries in a Caribbean population: a computed tomography based study. Springerplus.

[19] Natsis K, Paraskevas G, Panagouli E, Tsaraklis A, Lolis E, Piagkou M (2014). A morphometric study of multiple renal arteries in Greek population and a systematic review. Romanian J Morphology Emb Ryology.

[20] Za¤yapan R, Pelin C, Kürkçüo¤lu A (2009). A retrospective study on multiple renal arteries in Turkish population. Anat [Internet].

[21] Kok NFM, Dols LFC, Hunink MGM, Alwayn IPJ, Tran KTC, Weimar W (2008). Complex vascular anatomy in live kidney donation: imaging and consequences for clinical outcome. Transplantation.

[22] Toprak U, Erdoğan A, Gülbay M, Karademir M, Paşaoğlu E, Akar OE. Preoperative evaluation of renal anatomy and renal masses with helical CT, 3D-CT and 3D-CT angiography. Diagn Interventional Radiol. 2005. 15795842

[23] Ugurel MS, Battal B, Bozlar U, Nural MS, Tasar M, Ors F (2010). Anatomical variations of hepatic arterial system, coeliac trunk and renal arteries: an analysis with multidetector CT angiography. Br J Radiol.

[24] Budhiraja V, Rastogi R, Asthana AK (2010). Renal artery variations: embryological basis and surgical correlation. Rom J Morphol Embryol.

[25] Artz NS, Sadowski EA, Wentland AL, Djamali A, Grist TM, Seo S (2011). Reproducibility of renal perfusion MR imaging in native and transplanted kidneys using non-contrast arterial spin labeling. J Magn Reson Imaging.

[26] Trinquart L, Mounier-Vehier C, Sapoval M, Gagnon N, Plouin PF (2010). Efficacy of revascularization for renal artery stenosis caused by fibromuscular dysplasia: a systematic review and meta-analysis. Hypertension.

[27] Çiçekcibaşi AE, Ziylan T, Salbacak A, Şeker M, Büyükmumcu M, Tuncer I (2005). An investigation of the origin, location and variations of the renal arteries in human fetuses and their clinical relevance. Annals Anat.

[28] Yang C, Yang X, Wang S, Chen X, Liu K (2022). Primary Aldosteronism masked by accessory renal arteries: a Case Report. J Clin Med.

[29] Jamkar AA, Khan B, Joshi DS (2017). Anatomical study of renal and accessory renal arteries. Saudi J Kidney Dis Transpl.

[30] Worsley C, Knipe H. Accessory renal artery. Radiopaedia.org. Radiopaedia.org; 2013.

[31] Shete A, Shete A, Dube S, Field ADIJIRM. 2020 undefined. Sample size calculation in bio statistics with special reference to unknown population.

[32] Mustafa AYAE, Mohammed Ali Q, Elimam M, PRESENCE OF ACCESSORY RENAL, ARTERY IN SUDANESE PEOPLE (2016). Int J Anat Res.

[33] Salih MA, Hasan MA (2018). Renal artery morphology and anatomical variations among Sudanese subjects. Anat J Afr.

[34] Munnusamy K, Kasirajan SP, Gurusamy K, Raghunath G, Bolshetty SL, Chakrabarti S (2016). Variations in branching pattern of renal artery in kidney donors using CT angiography. J Clin Diagn Res.

[35] Regmi PR, Amatya I, Kayastha P, Poudel S, Suwal S, Ghimire RK (2020). Normal anatomy and variants of renal vasculature with multidetec-tor computed tomographyin a tertiary care hospital: a descriptive cross-sectional study. J Nepal Med Association.

[36] Aremu A, Igbokwe M, Olatise O, Lawal A, Maduadi K (2021). Anatomical variations of the renal artery: a computerized tomographic angiogram study in living kidney donors at a Nigerian kidney transplant center. Afr Health Sci.

[37] Abdessater M, Alechinsky L, Parra J, Malaquin G, Huot O, Bastien O (2022). Anatomical variations of the renal artery based on the surgeon’s direct observation: a French perspective. Morphologie.

[38] Gulas E, Wysiadecki G, Cecot T, Majos A, Stefańczyk L, Topol M (2016). Accessory (multiple) renal arteries– differences in frequency according to population, visualizing techniques and stage of morphological development.

[39] Tardo DT, Briggs C, Ahern G, Pitman A, Sinha S (2017). Anatomical variations of the renal arterial vasculature: an Australian perspective. J Med Imaging Radiat Oncol.

[40] Çlnar C, Türkvatan A (2016). Prevalence of renal vascular variations: evaluation with MDCT angiography. Diagn Interv Imaging.

[41] Costa HC, Moreira RJ, Fukunaga P, Fernandes RC, Boni RC, Matos AC. Anatomic variations in vascular and collecting systems of kidneys from deceased donors. In: Transplantation Proceedings. Transplant Proc; 2011. pp. 61–3. 10.1016/j.transproceed.2010.12.013 21335155

[42] Holden A, Smith A, Dukes P, Pilmore H, Yasutomi M. Assessment of 100 live potential renal donors for laparoscopic nephrectomy with multi-detector row helical CT. Vol. 237, Radiology. Radiology; 2005. pp. 973–80. 10.1148/radiol.2373041303 16304115

[43] Guan WH, Han Y, Zhang X, Chen DS, Gao ZW, Feng XS. Multiple renal arteries with renal cell carcinoma: preoperative evaluation using computed tomography angiography prior to laparoscopic nephrectomy. J Int Med Res. 2013;41(5):1705–15. 10.1177/0300060513491883 24003054

[44] Bordei P, Şapte E, Iliescu D. Double renal arteries originating from the aorta. Surg Radiol Anat. 2004;26(6):474–9. 10.1007/s00276-004-0272-9 15378279

[45] Aytac SK, Yigit H, Sancak T, Ozcan H (2003). Correlation between the diameter of the main renal artery and the presence of an accessory renal artery: Sonographic and angiographic evaluation. J Ultrasound Med.

[46] Rizzari MD, Suszynski TM, Gillingham KJ, Matas AJ, Ibrahim HN (2012). Outcome of living kidney donors left with multiple renal arteries. Clin Transpl.

